# Learning analytics: Survey data for measuring the impact of study satisfaction on students' academic self-efficacy and performance

**DOI:** 10.1016/j.dib.2019.104051

**Published:** 2019-05-23

**Authors:** Petros Kostagiolas, Charilaos Lavranos, Nikolaos Korfiatis

**Affiliations:** a Department of Archives, Library Science and Museology, Faculty of Information Science & Informatics, Ionian University, Greece; b Norwich Business School, University of East Anglia, UK

**Keywords:** Learning analytics, Study satisfaction, Academic self-efficacy, Academic performance, Information use, Undergraduate students

## Abstract

This paper presents learning analytics data for measuring the impact of study satisfaction on students' academic self-efficacy and performance. For this purpose, a specially designed questionnaire was developed and distributed across 124 undergraduate students. Preliminary analysis using descriptive statistics for items and confirmatory factor analysis is provided. The analysis provides evidence for the relation between students' satisfaction, self-efficacy, and academic performance, and evaluates the role of academic information resources in fulfilling students' information needs. These data are of importance for researchers and practitioners involved with budgetary decisions in academic collections as well as the influence of research specific (rather than training specific) information resources in student satisfaction.

**Table:** Specifications table

Subject area	Social Science
More specific subject area	Education
Type of data	Tables
How data was acquired	Hard copy questionnaire
Data format	Raw, Analyzed
Experimental factors	A qualitative pilot study was performed in the questionnaire development stage before being distributed to students. The students asked to self-assess their sense of academic satisfaction, self-efficacy, and performance. Moreover, they addressed their information resources usage, as well as the fulfillment of their information needs.
Experimental features	Data was collected using hard copy forms to all students eligible for participation. The response forms were collected by a volunteer student and given back to the researcher in a closed envelope. Consent was given by the school board and no personal identifiable information was required.
Data source location	Greece
Data accessibility	Data is included in this article
Related research article	P. Gkorezis, P. Kostagiolas, D. Niakas, Linking exploration to academic performance: The role of information seeking and academic self-efficacy, Library Management. 38 (2017) 404–414.

**Table:** 

**Value of the Data** • The data provided in this paper reveal the role of study satisfaction on students' academic self-efficacy and performance. • The dataset is among the very few available containing primary data dealing with the issue of the impact of study satisfaction on students' academic self-efficacy and performance. • The dataset can be utilized by other researchers in researching the impact of study satisfaction on students' academic self-efficacy and performance. It can provide significant value to those researchers interested in meta-analytic relations between student satisfaction and academic performance. • Researchers and practitioners can reproduce and extend this analysis by repeating the survey in different contexts, i.e., other countries, universities, specific student groups, etc.

## 1 Data

Learning analytics have become a subject of particular importance, especially considering the abundance of secondary data encompassing all aspects of student trajectories across an academic curriculum [1], [2]. Students' academic performance is of particular interest to higher education institutions internationally, in the view of the support services provided to complement academic services [3]. Understanding the factors that drive students' academic performance is becoming an important topic for researchers and education policymakers with several government initiatives been undertaken recently (e.g., UK government Teaching Excellence Framework - TEF^1^). As a result, the vital role of factors such as the academic environment, study habits, educational skills, and personality traits in optimizing students' academic performance is emphasized and acknowledged in the literature [4]. Nonetheless, there are scant datasets of primary data available to back up the influence of study satisfaction to students' academic self-efficacy and performance.

The complementary importance of primary data in that case relies to the multi-dimensional nature of student performance and the various metrics and methods available to quantify it. Therefore, the objective of this dataset is biforld. First it aims to provide raw survey data for measuring students' academic satisfaction, self-efficacy, and performance. Second it aims to provide evidence on the impact of study satisfaction on students' academic self-efficacy and performance. A hard copy questionnaire was developed and administered to students who attended an undergraduate course at a Greek regional university. An outline of basic insights using descriptive statistics and confirmatory factor analysis is provided in the sections that follow.

## 2 Experimental design, materials, and methods

The survey was conducted during the second semester of the academic year 2017–2018 and included a total number of 124 undergraduate students in Greece. Consent was given by the school board and the data collection procedure was compliant with the privacy policy of the University. Hard copy response forms were distributed to all students of the academic program and no sampling was performed. The distribution was done in classroom before lecture and the forms were collected by a volunteer student and provided back to the researcher in a closed envelope. Table 1 depicts the questionnaire elements, measurement types, and associated variable codes. More specifically, the specially designed questionnaire includes the following sections:

**Table 1:** Questionnaire elements, measurement types and associated variable codes.

Code	Question	Measurement Type
A1	Sex	Nominal (Categories: 1 = Male, 2 = Female)
A2	Age	Range
A3	Study Direction	Nominal (Categories: 1 = Archives, 2 = Library Science, 3 = Museology)
A4	Year of Study	Ordinal Scale (Categories: 1 = 1st Year, 2 = 2nd Year, 3 = 3rd Year, 4 = 4th Year, 5 = Extension)
A5	Familiarity with English	Ordinal Scale (Categories: 1 = Not at all, 2 = A little, 3 = Quite a bit, 4 = A lot, 5 = Very much)
B1	*I am confident about my ability to do my job*	
B2	*I am self-assured about my capabilities to perform my wok activities*	
B3	*I have mastered the skills necessary for my job*	
B4	*All in all, I am satisfied with my job*	
B5	*In general, I do not like my job*	
B6	*In general, I like working here*	
B7	*I perform tasks that are expected of me*	
B8	*I meet formal performance requirements of the job*	
B9	*I am involved in activities that are relevant to my yearly performance assessment*	
C1	*Scientific Databases (e.g., PubMed)*	
C2	*Scientific Journals (e.g., International Journal on Digital Libraries)*	
C3	*Encyclopedias, dictionaries and other reference works*	
C4	*E-learning system (e-class)*	
C5	*Electronic Portals and Websites (e.g., Thematic Portals of the University)*	
C6	*Social networking sites (e.g., Facebook, LinkedIn)*	
C7	*Search Engines (e.g., Google)*	
C8	*Communication with other University students*	
C9	*Contact Professor - Instructor*	
C10	*In general, I am satisfied with the use of information resources in my studies*	
D1	*In general, I am able to fulfill my information needs in my studies*	

Section A: Demographics: five (5) variables (sex; age; study line; year of study; familiarity with English).

Section B: Self-assessment of academic satisfaction, self-efficacy, and performance: nine (9) items obtained from established scales in the literature [5], [6].

Section C: Self-assessment of information resources usage: ten (10) variables (Scientific Databases; Scientific Journals; Encyclopedias, dictionaries and other reference works; E-learning system; Electronic Portals and Websites; Social networking sites; Search Engines; Communication with other University students; Contact with Professor – Instructor; and the general satisfaction with the information resources available. This was adapted from Ref. [7].

Section D: Self-assessment of information needs fulfillment: one (1) variable (*In general, I am able to fill my information needs in my studies*). This can also be considered an outcome variable.

All items were measured using a 5-point Likert type scale with options at 1 = “not at all”, 2 = “a little”, 3 = “quite a bit”, 4 = “a lot” and 5 = “very much”. Using Confirmatory Factor Analysis (CFA). Items from Sections A to C have been also utilized by other studies in the literature [8], [9], [10].

Table 2 presents the demographic characteristics of the responded students which comprise of sex, age, study direction, year of study, as well as familiarity with English. The gender distribution of the respondents shows that 17.1% are males; the average age is 21.47; while the study program specialization of the respondents is 43.2% Archives, 12.6% Library Science, and 44.1% Museology. Furthermore, the study year of the respondents is 35.8% 2nd, 27.5% 3rd, 24.2% 4th, and 11.7% extension, as well as their familiarity with English, is 9.6% a little, 35.7% quite a bit, 38.3% a lot, and 16.5% very much.

**Table 2:** Sample characteristics.

Gender (% of Sample)	
*Males*	17.1
**Age**	
*Mean (SD)*	21.47 (3.6)
**Study Direction** (% of Sample)	
*Archives*	43.2
*Library Science*	12.6
*Museology*	44.1
**Year of Study** (% of Sample)	
*1*st *Year*	0.8
*2*nd *Year*	35.8
*3*rd *Year*	27.5
*4*th *Year*	24.2
*Extension*	11.7
**Familiarity with English** (% of Sample)	
*A little*	
*Quite a Bit*	35.7
*A lot*	38.3
*Very Much*	16.5

In order to provide a meaningful structure and usefulness to other researchers, especially in relation with latent factors involved with the design of the questionnaire, we followed all the procedural remedies for confirmatory factor analysis discussed in Ref. [11]. Table 3 provides the results for item loadings and reliability changes for academic self-efficacy (E1), satisfaction (E2) and performance (E3).

**Table 3:** CFA loadings and reliability changes for academic self-efficacy, satisfaction and performance.

Factor	Item	Std. Loading (t-Value)	Alpha	Alpha if item removed	AVE
Academic Self-efficacy (E1)	B1	0.85 (18.36)	0.80	0.70	0.61
	B2	0.86 (18.99)		0.72	
	B3	0.61 (8.12)		0.85	
Academic Satisfaction (E2)	B4	0.77 (10.66)	0.81	0.59	0.50
	B5	0.70 (9.09)		0.68	
	Β6	0.64 (7.81)		0.73	
Academic Performance (E3)	B7	0.63 (8.39)	0.75	0.82	0.60
	B8	0.87 (17.69)		0.60	
	B9	0.81 (14.95)		0.76	

To evaluate cases of multicollinearity between factor items and outcome variables, the item-correlation matrix (Table 4) is provided and show no concerns (maximum inter-item correlation less than 0.70).

**Table 4:** Item correlation matrix.

	B1	B2	B3	B4	B5	B6	B7	B8	B9	C1	C2	C3	C4	C5	C6	C7	C8	C9	C10	D1
**B1**	1																			
**B2**	0.745^∗∗∗^	1																		
**B3**	0.552^∗∗∗^	0.500^∗∗∗^	1																	
**B4**	0.254^∗∗^	0.314^∗∗∗^	0.219^∗^	1																
**B5**	0.198^∗^	0.210^∗^	0.131	0.588^∗∗∗^	1															
**B6**	0.217^∗^	0.247^∗^	0.225^∗^	0.548^∗∗∗^	0.498^∗∗∗^	1														
**B7**	0.393^∗∗∗^	0.431^∗∗∗^	0.298^∗∗^	0.249^∗∗^	0.254^∗∗^	0.333^∗∗∗^	1													
**B8**	0.447^∗∗∗^	0.429^∗∗∗^	0.396^∗∗∗^	0.270^∗∗^	0.130	0.331^∗∗∗^	0.563^∗∗∗^	1												
**B9**	0.410^∗∗∗^	0.399^∗∗∗^	0.356^∗∗∗^	0.130	0.089	0.171	0.418^∗∗∗^	0.684^∗∗∗^	1											
**C1**	0.143	0.257^∗∗^	0.080	0.152	0.087	0.079	0.261^∗∗^	0.271^∗∗^	0.171	1										
**C2**	0.286^∗∗^	0.294^∗∗^	0.313^∗∗∗^	0.258^∗∗^	0.152	0.219^∗^	0.380^∗∗∗^	0.223^∗^	0.142	0.354^∗∗∗^	1									
**C3**	0.174	0.252^∗∗^	0.177	0.148	0.128	0.171	0.255^∗∗^	0.115	0.099	0.181	0.483^∗∗∗^	1								
**C4**	0.026	0.046	−0.044	0.161	0.157	0.078	0.077	−0.086	−0.087	−0.046	0.112	0.228^∗^	1							
**C5**	0.131	0.170	0.116	0.142	0.144	0.067	0.122	−0.030	0.091	0.302^∗∗^	0.187	0.373^∗∗∗^	0.226^∗^	1						
**C6**	−0.027	0.072	0.022	0.264^∗∗^	0.293^∗∗^	0.120	0.092	−0.017	0.037	0.085	0.108	0.051	0.298^∗∗^	0.382^∗∗∗^	1					
**C7**	−0.162	−0.087	−0.093	0.124	0.156	0.054	0.082	−0.175	−0.122	0.001	0.125	0.212^∗^	0.616^∗∗∗^	0.204^∗^	0.333^∗∗∗^	1				
**C8**	0.119	0.126	0.216^∗^	0.163	0.187	0.021	0.104	0.020	0.074	−0.028	−0.141	0.071	0.319^∗∗∗^	0.288^∗∗^	0.264^∗∗^	0.197^∗^	1			
**C9**	0.013	0.050	0.105	0.209^∗^	0.221^∗^	0.108	0.150	−0.009	0.042	0.025	0.149	0.369^∗∗∗^	0.289^∗∗^	0.185	0.210^∗^	0.270^∗∗^	0.354^∗∗∗^	1		
**C10**	0.229^∗^	0.288^∗∗^	0.202^∗^	0.291^∗∗^	0.319^∗∗∗^	0.198^∗^	0.346^∗∗∗^	0.226^∗^	0.134	0.239^∗^	0.356^∗∗∗^	0.476^∗∗∗^	0.405^∗∗∗^	0.401^∗∗∗^	0.358^∗∗∗^	0.410^∗∗∗^	0.235^∗^	0.434^∗∗∗^	1	
**D1**	0.289^∗∗^	0.297^∗∗^	0.230^∗^	0.206^∗^	0.157	0.027	0.240^∗^	0.154	0.259^∗∗^	0.138	0.296^∗∗^	0.128	0.123	0.240^∗^	0.206^∗^	0.183	0.088	0.214^∗^	0.376^∗∗∗^	1

Computed correlation used pearson-method with listwise-deletion., Note *p < 0.05, **p < 0.01, ***p < 0.001.

The factor correlation matrix between the three identified factors (E1, E2, E3) and the two outcome variables of interest (C10, D1) is provided in Table 5. For the factor structure, the square root of the average variance extracted from CFA is reported in the diagonal and is higher than the reported factor correlation, thus satisfying discriminant validity using the Fornell-Larcker criterion [12].

**Table 5:** Factor Correlation Matrix with outcome variables (C10, D1).

	E1	E2	E3	C10	D1
E1	***0.78***				
E2	0.449	***0.70***			
E3	0.629	0.291	***0.77***		
C10	0.357	0.393	0.269	–	
D1	0.344	0.377	0.274	0.413	–

Note: The bold italic numbers signifies the square root of AVE (Average Variance Extracted) values for the factors E1, E2, and E3. Evidently, the discriminant validity of the scale is established since the abovementioned values are greater than the reported factor correlations.

This dataset shows that factors such as study satisfaction combined with a sense of self-efficacy may act as an essential mechanism for improving students' academic performance and the overall satisfaction with their studies. On the other hand, academic information resources usage assessment is continually improving affecting students' academic performance as well, especially in cases of research-oriented instruction. This dataset can help researchers and institutions (universities, scholars, students, etc.) to comprehend the important role and the impact of academic program satisfaction to students' academic self-efficacy and performance. With the ongoing trend in the deployment of learning analytics, the outcomes showcase that potential investments in academic information services to support the educational and research process may result in an improvement in students' academic performance. The latter is important to administrators and policymakers considering the important budgetary decisions related to the allocation of funds for academic subscriptions of teaching and learning vs. research-oriented material. Enrichment activities of the presented data could also target areas of improvement related to assessment and other primary sources of academic achievement [13].

## Funding resources

This research did not receive any specific grant from funding agencies in the public, commercial, or not-for-profit sectors.

## Footnotes

1 UK Government – Teaching Excellence Framework (Year 2) Specification. Available at https://www.gov.uk/government/publications/teaching-excellence-and-student-outcomes-framework-specification.
