# Supplementary material for: Learning analytics: Survey data for measuring the impact of study satisfaction on students' academic self-efficacy and performance
Source: Data Brief. 2019 May 23;25:104051. doi: 10.1016/j.dib.2019.104051 (PMC6562179; doi:10.1016/j.dib.2019.104051)
Supplement: Multimedia component 1 [file mmc1.docx]

**Conflict of Interest Declaration**

All authors declare that no conflict of interest exists with the attached data and manuscript.

Dr Petros Kostagiolas, Dr Charilaos Lavranos, Dr Nikolaos Korfiatis
